# Carcinoma-specific expression of P2Y_11_ receptor and its contribution in ATP-induced purinergic signalling and cell migration in human hepatocellular carcinoma cells

**DOI:** 10.18632/oncotarget.16191

**Published:** 2017-03-14

**Authors:** Madiha Khalid, Lucie Brisson, Menahil Tariq, Yunjie Hao, Roseline Guibon, Gaëlle Fromont, Sharifah Alawieyah Syed Mortadza, Fatema Mousawi, Sobia Manzoor, Sébastien Roger, Lin-Hua Jiang

**Affiliations:** ^1^ School of Biomedical Sciences, Faculty of Biological Sciences, University of Leeds, Leeds, UK; ^2^ Atta-ur-Rahman School of Applied Biosciences, National University of Science and Technology, Islamabad, Pakistan; ^3^ Inserm UMR1069, Nutrition, Croissance et Cancer, Université François-Rabelais de Tours, Tours, France; ^4^ Centre Hospitalo-Universitaire de Tours, Tours, France; ^5^ Institut Universitaire de France, Paris Cedex 05, France; ^6^ Department of Physiology and Neurobiology, Xinxiang Medical University, Xinxiang, P. R. China; ^7^ Sino-UK Joint Laboratory of Brain Function and Injury, Xinxiang Medical University, Xinxiang, P. R. China

**Keywords:** HCC cells, extracellular ATP, P2Y_11_ receptor, cytosolic Ca^2+^, cell migration

## Abstract

Extracellular ATP-induced Ca^2+^ signalling is critical in regulating diverse physiological and disease processes. Emerging evidence suggests high concentrations of extracellular ATP in tumour tissues. In this study, we examined the P2 receptor for ATP-induced Ca^2+^ signalling in human hepatocellular carcinoma (HCC) cells. Fura-2-based measurements of the intracellular Ca^2+^ concentration ([Ca^2+^]_i_) showed that extracellular ATP induced an increase in the [Ca^2+^]_i_ in human HCC Huh-7 and HepG2 cells. NF546, a P2Y_11_ receptor agonist was equally effective in inducing an increase in the [Ca^2+^]_i_. In contrast, agonists for the P2X receptors (αβmeATP and BzATP), P2Y_1_ receptor (MRS2365) or P2Y_2_ receptor (MRS2768) were ineffective. In addition, ATP/NF546-induced increases in the [Ca^2+^]_i_ were strongly inhibited by treatment with NF340, a P2Y_11_ receptor antagonist. Immunofluorescent confocal imaging and western blotting analysis consistently demonstrated the P2Y_11_ receptor expression in Huh-7 and HepG2 cells. Transfection with P2Y_11_-specific siRNA attenuated the P2Y_11_ receptor protein expression level and also reduced NF546-induced increase in the [Ca^2+^]_i_. Importantly, immunohistochemistry revealed that the P2Y_11_ receptor was expressed at very high level in human HCC tissues and, by contrast, it was barely detected in normal liver tissues. Trans-well cell migration assay demonstrated that ATP and NF546 induced concentration-dependent stimulation of Huh-7 cell migration. Treatment with NF340 prevented ATP-induced stimulation of cell migration. Taken together, our results show carcinoma-specific expression of the P2Y_11_ receptor and its critical role in mediating ATP-inducing Ca^2+^ signalling and regulating cell migration in human HCC cells.

## INTRODUCTION

Hepatocellular carcinoma (HCC) is the primary liver cancer; poor prognosis and ineffective treatment of HCC with currently available anti-cancer treatments have made it to be one of the leading and most deadly causes of cancer-related mortality, with the 5-year survival rate being less than 15% [1–4]. The global incidence of HCC, while exhibiting noticeable regional variations, has been reported to increase in the recent past and is anticipated to continue to rise in the coming years [3, 4]. While several disease risk factors including aging, genetics, infection and lifestyle such as smoking and alcohol have been identified to contribute to the development of HCC, it is much less well-understood with respect to the underlying molecular mechanisms. A mechanistic understanding of the pathogenesis and progression of HCC is of great value towards identifying disease biomarkers and drug targets for development of new diagnosis and effective treatments.

The microenvironment in tumour tissues is highly hypoxic, a condition that is well-documented to stimulate release of intracellular ATP [5–7]. *In vivo* imaging provides clear evidence to show that pericellular ATP can reach hundreds of micro-molar concentrations at the tumour sites but remains almost undetectable in normal tissues [6, 7]. It has been well established that extracellular ATP interacts with ligand-gated ion channel P2X receptors and G-protein-coupled P2Y receptors on the cell surface to induce autocrine and paracrine signalling [8–11]. There are seven mammalian P2X receptor proteins or subunits (P2X1-P2X7) that can assemble into homo/hetero-trimeric P2X receptors [12]. ATP activates all P2X receptors, albeit with different potency [13], that form an ion-conducting pathway across the plasma membrane that allows passage of cations including Ca^2+^. There are eight mammalian P2Y receptors that are activated by various extracellular nucleotides such as ATP, ADP, UTP and UDP [14]. ATP activates the human P2Y_1_, P2Y_2_ and P2Y_11_ receptors that are mainly coupled to G_α,q/11_ and thus their activation stimulates phospholipase C (PLC) and subsequent generation of IP_3_, which in turns activates the IP_3_ receptor (IP_3_R) in the endoplasmic reticulum (ER) to mediate ER Ca^2+^ release [14]. Therefore, ATP can elevate the intracellular Ca^2+^ concentrations ([Ca^2+^]_i_) via the P2X receptor-mediated extracellular Ca^2+^ influx or the P2Y receptor-PLC-IP_3_R signalling pathway leading to internal Ca^2+^ release. Mammalian cells express multiple P2X and P2Y receptors often in a cell type-specific manner [8, 9] that play a role in a diversity of physiological functions and pathological processes, including cancers [15–19]. Extracellular ATP has been reported to influence cancer cell functions, particularly cancer cell metastasis which is a key process responsible for the high mortality [20]. For example, recent studies of various types of cancer cells have shown that ATP-induced purinergic signalling regulates cancer cell migration, proliferation and survival via the P2X7 receptor [21–32] or P2Y_2_ receptor [33–37]. There is evidence to indicate mRNA and/or protein expression of the P2Y_1_ and P2Y_2_ receptors in primary and immortalized human normal hepatocytes, primary human HCC cells and immortal human HCC cells (e.g., Huh-7, HepG2 and BEL-7404) [37–39], and the P2X4 and P2X7 receptors in HepG2 cells, rat and mouse hepatocytes and rat HCC cells [38]. Further studies demonstrated that activation of the P2Y_2_ receptor leads to ATP-induced increase in the [Ca^2+^]_i_ in human normal hepatocytes and human HCC cells [37, 38]. In addition, the P2Y_2_ receptor expression is upregulated in human HCC cells and genetic suppression of the P2Y_2_ receptor expression inhibits human HCC cell migration [37]. In contrast, a separate study showed functional expression of the P2X4 receptor and possibly the P2X7 receptor in rat and mouse hepatocytes and rat HCC cells [39]. Thus, different P2X and P2Y receptors have been reported in rodent and human hepatocytes and HCC cells. In the present study, we provide pharmacological, functional and genetic evidence to support the P2Y_11_ receptor in ATP-induced Ca^2+^ signalling in human HCC cells, reveal strong HCC-specific P2Y_11_ receptor expression, and propose their involvement in HCC cell migration.

## RESULTS

### ATP induces an increase in the [Ca^2+^]_i_ in Huh-7 cells

**Figure 1:**
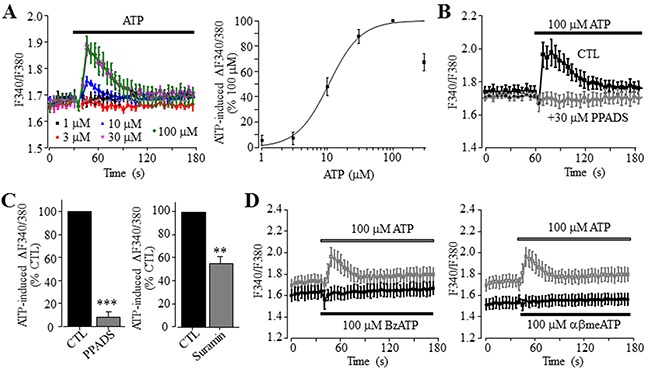
ATP induces concentration-dependent increase in the [Ca^2+^]_i_ in Huh-7 cells **(A)** *Left*, representative recordings of Ca^2+^ responses induced by 1-100 μM ATP, with six wells of cells for each concentration. *Right*, ATP concentration-peak Ca^2+^ response curve, constructed by expressing the responses induced by 1-300 μM ATP as % of that induced by 100 μM ATP. Each data point represents three independent experiments. The smooth line represents the least squared fit to Hill equation with EC_50_ and Hill coefficient of 11 μM ATP and 1.8, respectively. **(B)** Representative recordings of Ca^2+^ responses induced by 100 μM ATP, in control cells or cells pre-treated with 30 μM μM PPADS, with six wells of cells for each case. **(C)** Summary of ATP-induced peak increase in the [Ca^2+^]_i_ in control and cells treated with indicated concentrations of 30 μM PPADS (left panel) and 30 μM suramin (right panel), expressed as % of that in control cells, from three independent experiments. **, p < 0.01; ***, p < 0.001. **(D)** Representative recordings of Ca^2+^ responses induced by 100 μM BzATP (left panel) or 100 μM αβmeATP (right panel) and 100 μM ATP (in grey), with six wells of cells for each case. Such results were observed in three independent experiments.

We began with measuring intracellular Ca^2+^ responses to ATP in human HCC Huh-7 cells, using fura-2 based ratiometry and FLEX-station. In the extracellular Ca^2+^-containing solution, ATP applied at 1-300 μM induced increases in the [Ca^2+^]_i_ in a concentration-dependent manner (Figure 1A). ATP-induced increase in the [Ca^2+^]_i_ reached the maximum at 100 μM, and slightly reduced at 300 μM ATP (Figure 1A) probably due to receptor desensitization. Fitting the data to Hill equation yielded an EC_50_ of 11 μM and Hill coefficient of 1.8 (Figure 1A). Pre-treatment with 30 μM PPADS or suramin, two P2 receptor generic antagonists, strongly inhibited ATP-induced Ca^2+^ responses (Figure 1B and 1C). These results provide the first indication that ATP can increase the [Ca^2+^]_i_ in Huh-7 cells via the P2 receptor.

As introduced above, activation of the P2X ionotropic receptor can increase the [Ca^2+^]_i_ as a result of extracellular Ca^2+^ influx. Protein expression of the P2X7 receptor in Huh-7 cells was previously reported [40] and confirmed using immunocytochemistry (Supplementary Figure 1). However, application of 100 and 300 μM 2’,3’-O-(4-benzoylbenzoyl)-ATP (BzATP), an agonist exhibiting a greater potency than ATP at the human P2X7 receptor and also activating other P2X receptor [18], completely failed to induce any detectable Ca^2+^ response (Figure 1D). This suggests poor functional expression of the P2X7 receptor. We also examined αβmethyleneATP (αβmeATP), which potently activates the human P2X receptors containing P2X1, P2X3 or P2X5 subunit [11]. There was no discernible Ca^2+^ response to 100 μM αβmeATP (Figure 1D). A previous study showed functional expression of the P2X4 receptor in rodent HCC cells [40]. Treatment with 10 μM 5-BDBD, a selective P2X4 receptor antagonist with a submicromolar potency [11], however, did not inhibit ATP-induced Ca^2+^ response (Supplementary Figure 2A), suggesting no major function of the P2X4 receptor at the plasma membrane of Huh-7 cells.

### P2Y_11_ receptor plays a key role in ATP-induced increase in the [Ca^2+^]_i_ in Huh-7 cells

**Figure 2:**
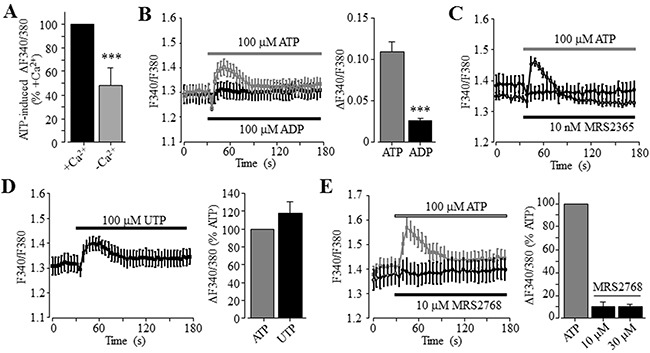
No major role of the P2Y_1_ and P2Y_2_ receptors in ATP-induced increase in the [Ca^2+^]_i_ in Huh-7 cells **(A)** Summary of 100 μM ATP-induced peak increase in the [Ca^2+^]_i_ in the extracellular Ca^2+^-containing and Ca^2+^-free solutions, expressed as % of that induced by ATP in the extracellular Ca^2+^-containing solution, from three independent experiments. **(B)** *Left*, representative recordings of Ca^2+^ responses induced by 100 μM ATP (grey) and ADP (black) in the extracellular Ca^2+^-containing solutions, with six wells of cells for each case. *Right*, summary of the peak increase in the [Ca^2+^]_i_.***, p < 0.001 (A and B). **(C)** Representative recordings of Ca^2+^ responses induced by 100 μM ATP (grey) and 10 μM MRS2365 (black) in the extracellular Ca^2+^-containing solution, from six wells of cells for each concentration. **(D)** *Left*, representative recordings of Ca^2+^ responses induced by 100 μM UTP in the extracellular Ca^2+^-containing solution with six wells of cells for each case. *Right*, summary of UTP-induced peak increase in the [Ca^2+^]_i_, expressed as % of that induced by 100 μM ATP, from three independent experiments. **(E)** *Left*, representative recordings of the Ca^2+^ responses induced by 10 μM MRS2768 (black) and 100 μM ATP (grey) in the extracellular Ca^2+^-containing solution with six wells of cells for each case. *Right*, summary of MRS2768-induced peak increase in the [Ca^2+^]_i_, expressed as % of that induced by 100 μM ATP, from three independent experiments.

**Figure 3:**
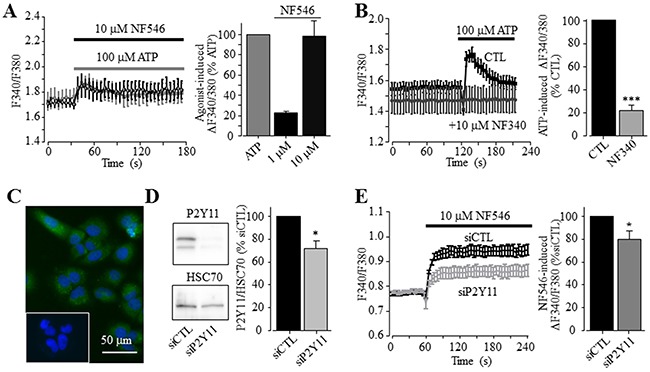
A key role of the P2Y_11_ receptor in ATP-induced increase in the [Ca^2+^]_i_ in Huh-7 cells **(A)** *Left*, representative recordings of Ca^2+^ responses induced by 10 μM NF546 (black) and 100 μM ATP (grey) in the extracellular Ca^2+^-containing solution with six wells of cells for each case. *Right*, summary of the peak increase in the [Ca^2+^]_i_ induced by 1 and 10 μM NF546, expressed as % of that induced by 100 μM ATP, from three independent experiments. **(B)** *Left*, representative recordings of Ca^2+^ responses induced by 100 μM ATP in control cells or cells pre-treated with 10 μM NF340, with six wells of cells for each case. *Right*, summary of ATP-induced peak increases in the [Ca^2+^]_i_ in control or NF340- treated cells, expressed as % of that in control cells, from three independent experiments. ***, p < 0.001. **(C)** Representative confocal images showing P2Y_11_ immunostaining. Cells were countstained with DAPI. The insert shows control cells stained only with the secondary antibody. Similar results were observed in two independent experiments. **(D)** *Left*, representative western blot analysing P2Y_11_ receptor expression in cells transfected with control and P2Y_11_-specific siRNA (siCTL and siP2Y11). *Right*, summary of mean P2Y_11_ protein expression, normalized to the HSC70 protein level and presented as % of the value in cells transfected with siCTL, from six independent experiments. *, p < 0.05. **(E)** *Left*, representative recordings of Ca^2+^ responses induced by 10 μM NF546 in cells transfected with indicate siRNA, with 4 wells of cells for each case. *Right*, summary of ATP-induced peak increases in the [Ca^2+^]_i_ in cells transfected with siCTL and siP2Y11, expressed as % of that in cells transfected with siCTL, from six independent experiments.

To examine the role of P2Y receptors in ATP-induced increase in the [Ca^2+^]_i_, we determined ATP-induced Ca^2+^ responses in the extracellular Ca^2+^-free solution, a widely-used experimental condition to determine Ca^2+^ release from internal stores. ATP was effective in inducing significant increase in the [Ca^2+^]_i_ in the extracellular Ca^2+^-free solution, albeit the amplitude of ATP-induced Ca^2+^ response was lower than that obtained in the presence of extracellular Ca^2+^ (Figure 2A). Taken together, these observations clearly support functional expression of ATP-sensitive P2Y receptors. To elaborate which particular P2Y receptor is involved in mediating ATP-induced Ca^2+^ response, we further examined several P2Y type selective agonists. Exposure to 100 μM ADP, an agonist that preferentially activates the P2Y_1_ receptor, induced very small but detectable Ca^2+^ responses in both extracellular Ca^2+^-free and Ca^2+^-containing solutions (Figure 2B and Supplementary Figure 2B). Moreover, application of 10 nM MRS2365, a selective P2Y_1_ receptor agonist, was ineffective in inducing an increase in the [Ca^2+^]_i_ (Figure 2C). These results consistently indicate no major role for the P2Y_1_ receptor in ATP-induced Ca^2+^ response. UTP, an agonist at the P2Y_2_ receptor as well as at the P2Y_4_ and P2Y_6_ receptors, was equally effective as ATP in elevating the [Ca^2+^]_i_ in the extracellular Ca^2+^-containing contains (Figure 2D), as previously reported [38]. These results are consistent with the pharmacological properties of the P2Y_2_ receptor, which was shown to be expressed in both human hepatocytes and HCC cells [37, 38]. Thus, it was surprising to find that exposure to 10 and 30 μM MRS2768, a selective P2Y_2_ receptor agonist, failed to elevate the [Ca^2+^]_i_ in the extracellular Ca^2+^-containing solution (Figure 2E). Such an observation was made using two batches of MRS2768 from different vendors (see Materials and Methods). In contrast, application of 1 and 10 μM NF546, a selective P2Y_11_ receptor agonist, concentration-dependently increased the [Ca^2+^]_i_ in the extracellular Ca^2+^-containing solution, and the Ca^2+^ response amplitude induced by 10 μM NF546 was virtually the same as that induced by 100 μM ATP (Figure 3A). Moreover, pre-treatment with 10 μM NF340, a selective P2Y_11_ receptor antagonist, almost completely abolished ATP-induced increase in the [Ca^2+^]_i_ in the extracellular Ca^2+^-containing solution (Figure 3B). Immunofluorescent confocal imaging demonstrated expression of the P2Y_11_ receptor in Huh-7 cells (Figure 3C and Supplementary Figure 1). Similarly, western blotting analysis supports protein expression of the P2Y_11_ receptor, which was attenuated by transfection with P2Y_11_-specific siRNA (Figure 3D). Consistently, siRNA-mediated knockdown of the P2Y_11_ receptor expression resulted in significantly smaller Ca^2+^ response to NF546 (Figure 3E). Taken together, these pharmacological, biochemical and genetic results provide consistent evidence to support expression of the P2Y_11_ receptor and its key role in mediating ATP-induced Ca^2+^ response in Huh-7 cells.

### P2Y_11_ receptor also contributes in ATP-induced Ca^2+^ signalling in HepG2 cells

**Figure 4:**
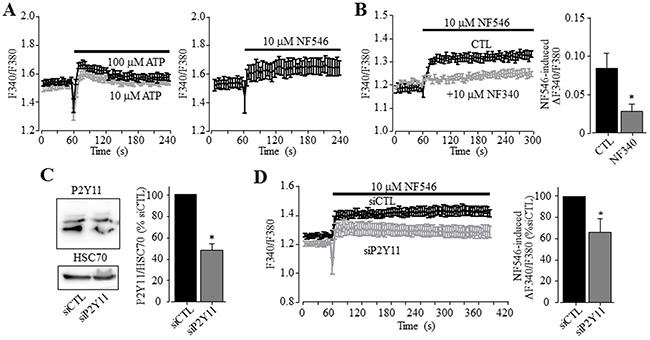
A role of the P2Y_11_ receptor in ATP-induced increase in the [Ca^2+^]_i_ in HepG2 cells **(A)** Representative recordings of Ca^2+^ responses induced by 10 μM (grey) and 100 μM ATP (black) (left panel) and 10 uM NF546 (right panel) in the extracellular Ca^2+^-containing solution with 4 wells of cells for each case. **(B)** *Left*, representative recordings of the Ca^2+^ responses induced by 10 μM NF546 in control cells or cells pre-treated with 10 μM NF340, with 4 wells of cells for each case. *Right*, summary of NF546-induced peak increase in the [Ca^2+^]_i_ in control or NF340-treated cells, from three independent experiments. **(C)** *Left*, representative western blot showing P2Y_11_ receptor expression in cells transfected with control and P2Y_11_-specific siRNA (siCTL and siP2Y11). *Right*, summary of the mean P2Y_11_ protein expression, normalized to HSC70 protein and presented as % of the value in cells transfected with siCTL, from 8 independent experiments. **(D)** *Left*, representative recordings of Ca^2+^ responses induced by 10 μM NF546 in cells transfected with indicate siRNA, with 4 wells of cells for each case. *Right*, summary of ATP-induced peak increase in the [Ca^2+^]_i_ in cells transfected with siCTL and siP2Y11, expressed as % of that in cells transfected with siCTL, from three independent experiments. *, p < 0.05.

To further investigate whether the P2Y_11_ receptor plays a more general role in mediating ATP-induced Ca^2+^ signalling in human HCC cells, we measured Ca^2+^ responses to ATP and NF546 in HepG2 cells. Like in Huh-7 cells, 10-100 μM ATP induced noticeable increase in the [Ca^2+^]_i_ in HepG2 cells (Figure 4A). Similarly, 10 μM NF546 was also effective (Figure 4B). Treatment with NF340 significantly attenuated NF546-induced Ca^2+^ response (Figure 4B). Western blotting showed protein expression of the P2Y_11_ receptor, with its expression level being strongly reduced in HepG2 cells after transfection with P2Y_11_-specific siRNA (Figure 4C). Such siRNA-mediated knockdown of the P2Y_11_ receptor expression significantly decreased NF546-induced Ca^2+^ response (Figure 4D).

### P2Y_11_ expression is abundantly expressed in human HCC but not normal live tissues

**Figure 5:**
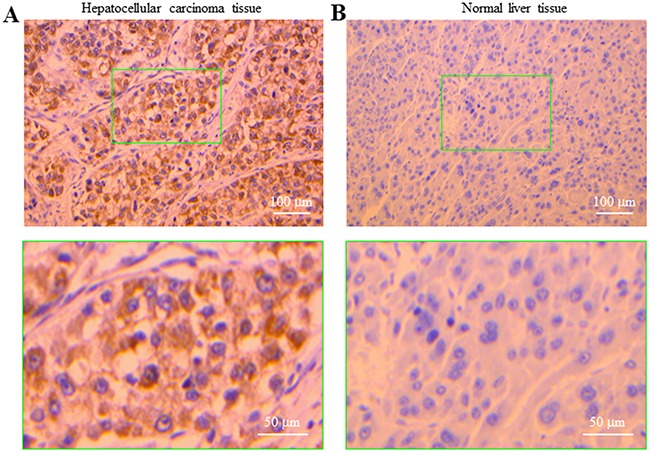
Expression of the P2Y_11_ receptor in human hepatocellular carcinoma and normal live tissues Immunostaining of the P2Y_11_ receptor in human HCC **(A)** and normal liver tissues **(B)**, counterstained with haematoxylin. The bottom panels show enlarged images of the areas in the green rectangle in the top panels.

The findings described above in two human HCC cells prompted us to investigate whether the P2Y_11_ receptor is expressed in primary human HCC tissues and, furthermore, whether the P2Y_11_ receptor expression in human HCC tissues differs from that in normal liver tissues. Immunohistochemistry showed abundant expression of the P2Y_11_ receptor, mainly located closely to the plasma membrane, in human HCC tissues (Figure 5A). In striking contrast, there was no immunostaining of the P2Y_11_ receptor in normal liver tissues (Figure 5B). These results provide evidence to support the P2Y_11_ receptor is expressed in human HCC tissues as well as human HCC cells and, furthermore, such P2Y_11_ receptor expression appears HCC-specific.

### ATP and NF546 stimulate Huh-7 cell migration

**Figure 6:**
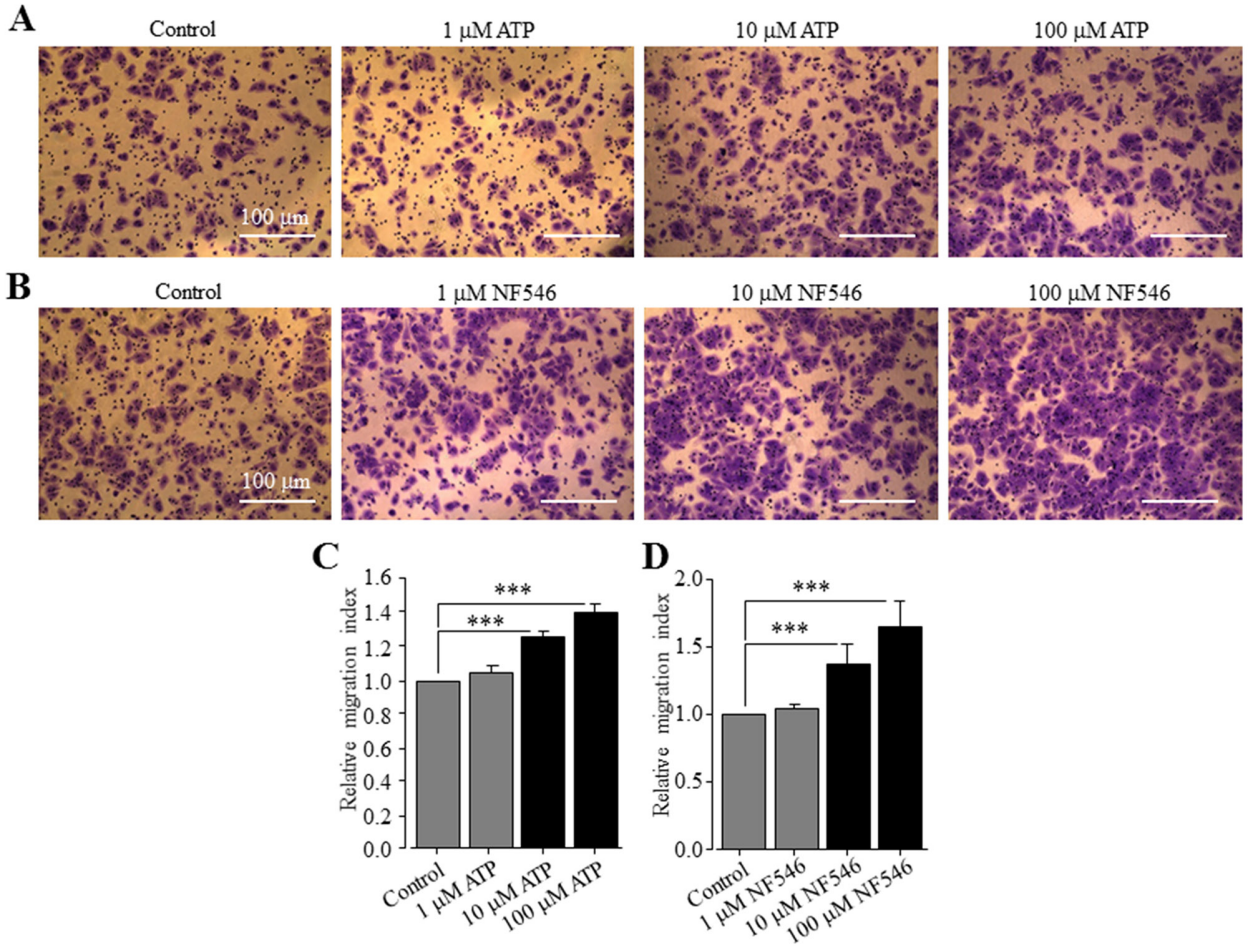
ATP stimulates Huh-7 cell migration via activating the P2Y_11_ receptor **(A-B)** Representative crystal violet staining images showing cell migration under basal condition and in the presence of indicated concentrations of ATP (A) and NF546 (B) in trans-well assays. **(C-D)** Summary of the effects of different concentrations of ATP (C) and NF546 (D) on cell migration in three independent experiments, respectively. ***, p < 0.001.

**Figure 7:**
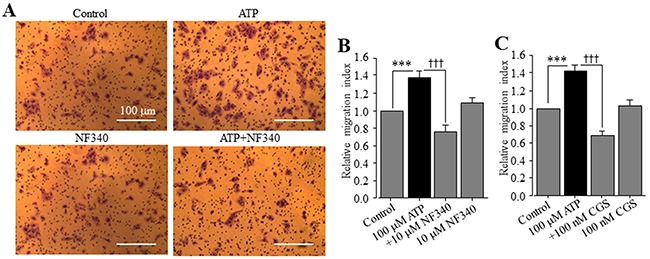
P2Y_11_ and adenosine receptors are involved in ATP-stimulated Huh-7 cell migration **(A)** Representative crystal violet staining images showing cell migration under the basal (control) condition and in the presence of 100 μM ATP alone, 10 μM NF340 alone, and 100 μM ATP and 10 μM NF546 (ATP+NF340) in trans-well assays. **(B-C)** Summary of the effects of treatment with NF340 (B) and 100 nM CGS15943 (C) on ATP-induced increase in cell migration from three independent experiments. ***, †††, p < 0.001.

In a recent study, ATP is shown to promote cell migration of native human HCC cells and HepG2 and BEL-7404 cells, and activation of the P2Y_2_ receptor is proposed to be critical in mediating ATP-induced cell migration [37]. This recent study has not examined the expression of the P2Y_11_ receptor and its role in ATP-induced stimulation of HCC cell migration, and also has not determined ATP-induced effect on Huh-7 cell migration. We thus performed the trans-well chamber assay to investigate whether extracellular ATP stimulated Huh-7 cell migration and if so, whether this could be reproduced using the specific P2Y_11_ receptor agonist NF546. ATP applied at 1, 10 and 100 μM resulted in concentration-dependent stimulation of cell migration (Figure 6A). The increase in cell migration by 10 and 100 μM ATP reached the significant level (Figure 6C). Treatment with 1, 10 and 100 μM NF546 also stimulated cell migration in a concentration-dependent manner (Figure 6B and 6D). At the same concentrations, NF546 induced slightly greater increase in cell migration than ATP (c.f. Figure 6C and 6D). Treatment with ATP at 100 μM induced no detectable cell death (Supplementary Figure 3). Taken together, these results provide clear evidence to support a critical role for the P2Y_11_ receptor in driving ATP-induced stimulation of Huh-7 cell migration. We also examined the effect of NF340 on ATP-induced stimulation of Huh-7 cell migration. Treatment with 10 μM NF340 completely blocked ATP-induced stimulation of cell migration with no significant effect on cell migration under the basal condition, providing further evidence to support that P2Y_11_ receptor activation is critical in stimulating cell migration by ATP (Figure 7A and 7B). It is well known that ATP is metabolically unstable and can be rapidly metabolized to ADP and further to adenosine by ecto-enzymes [40]. Adenosine also acts as an extracellular signalling molecule via activating G-protein-coupled adenosine receptors on the cell surface [8]. Activation of the adenosine receptors has been shown in a very recent study to be significantly involved in ATP-induced regulation of breast cancer cell migration [29]. The role of adenosine receptors in ATP-induced stimulation of HCC cell migration has not been investigated in previous studies. Therefore, we examined the effect of CGS15943, a generic antagonist for adenosine receptors, on ATP-induced stimulation of Huh-7 cell migration. Intriguingly, treatment with 100 nM CGS15943 was also effective in preventing ATP-induced increase in cell migration with no significant effect on cell migration under the basal condition (Figure 7A and 7C), suggesting a substantial role for adenosine receptors in ATP-induced stimulation in HCC cell migration.

## DISCUSSION

The present study provides pharmacological and genetic evidence that consistently supports functional expression of the P2Y_11_ receptor as a critical mechanism in mediating ATP-induced Ca^2+^ signalling and stimulating cell migration in human HCC cells and, importantly, provide evidence to suggest HCC-specific P2Y_11_ receptor expression.

We first showed that ATP induced robust increases in the [Ca^2+^]_i_ in Huh-7 cells (Figure 1A). ATP-induced Ca^2+^ responses were sensitive to inhibition by PPADS and suramin (Figure 1B and 1C). In addition, ADP was much less effective than ATP in elevating the [Ca^2+^]_i_ (Figure 2B). Overall, these results are consistent with those reported in an early study of Huh-7 cells [38] and also with those in a recent study of native human HCC cells and HepG2 and BEL-7404 cells [37]. In the present study, the P2Y_1_ receptor agonist MRS2365 was ineffective in inducing an increase in the [Ca^2+^]_i_ (Figure 2C), largely ruling out a major role of the P2Y_1_ receptor, despite its mRNA expression being previously reported in Huh-7 cells [38]. In Huh-7 cells, UTP was as potent as ATP in elevating the [Ca^2+^]_i_ (Figure 2D). Such results could be simply interpreted to indicate involvement of the P2Y_2_ receptor [38]. Indeed, a recent study shows that shRNA-mediated knockdown of the P2Y_2_ receptor reduced ATP-induced increase in the [Ca^2+^]_i_ in HepG2 and BEL-7404 cells, supporting functional expression of the P2Y_2_ receptor in these cells [37]. However, the present study found that the P2Y_2_ receptor agonist MRS2768 failed to induce any significant increase in the [Ca^2+^]_i_ in Huh-7 cells (Figure 2E). We examined expression of the P2Y_11_ receptor, another ATP-sensitive P2Y receptor and its role in ATP-induced Ca^2+^ responses in Huh-7 cells, which was not investigated in previous studies. Consistently, P2Y_11_ receptor agonist NF546 was equally effective as ATP in eliciting robust Ca^2+^ responses in Huh-7 cells (Figure 3A). Moreover, pre-treatment with P2Y_11_ receptor specific antagonist NF340 abrogated ATP-induced Ca^2+^ response (Figure 3B). Huh-7 cells showed positive P2Y_11_ receptor protein expression as examined by immunofluorescent confocal imaging (Figure 3C) and western blotting (Figure 3D). SiRNA-mediated knockdown of the P2Y_11_ receptor protein expression (Figure 3D) significantly attenuated NF546-induced increase in the [Ca^2+^]_i_ in Huh-7 cells (Figure 3E). Similarly, both ATP and NF546 were effective in evoking Ca^2+^ responses in HepG2 cells (Figure 4A) and NF546-induced increase in the [Ca^2+^]_i_ was inhibited by NF340 (Figure 4B). The P2Y_11_ receptor protein in HepG2 cells was also detected by western blotting (Figure 4C). Both the P2Y_11_ receptor protein expression level and NF546-induced Ca^2+^ response were significantly reduced by P2Y_11_-specific siRNA (Figure 4C and 4D). Collectively, our results provide strong evidence to indicate that the P2Y_11_ receptor is critical in ATP-induced Ca^2+^ response in human HCC cells.

ATP-induced Ca^2+^ responses in the extracellular Ca^2+^-free solution were significantly smaller than those in the extracellular Ca^2+^-containing solution (Figure 2A). This could result from two distinct molecular mechanisms. The first potential mechanism is that reduction in the ER Ca^2+^ level, due to Ca^2+^ release following activation of the P2Y_11_-G_α,q/11_-PLC-IP_3_R signalling pathway, activates the store-operated Ca^2+^ entry. Such Ca^2+^ signalling mechanism has been recently shown to exist in native human HCC cells and HepG2 and BEL-7404 cells [37]. The second and alternative mechanism is the P2X receptor that can also mediates extracellular Ca^2+^ influx. Previous studies, performed in rat HCC cells, showed that BzATP induced large fast-desensitizing inward currents using patch-clamp recording and a rapid extracellular Ca^2+^ influx using Ca^2+^ imaging, and the P2X4 receptor and possibly the P2X7 receptor were thought to mediate such responses [39]. While suramin and PPADS were effective in inhibiting ATP-induced Ca^2+^ responses (Figure 1B and 1C), the P2X4 receptor selective antagonist 5-BDBD resulted in no inhibition (Supplementary Figure 2A). In the present study, we confirmed protein expression of the P2X7 receptor in Huh-7 cells (Figure 3C and Supplementary Figure 1). However, there was no Ca^2+^ response to BzATP (Figure 1D), which is known to activate the P2X7 and other P2X receptors including the P2X4 receptor [13]. Exposure to αβmeATP also induced no increase in the [Ca^2+^]_i_ (Figure 1D), suggesting no expression of functional P2X receptors containing the P2X1, P2X3 or P2X5 subunit. While further studies are required to examine the potential contribution of P2X2 and P2X6 receptors, it is clear from the present study that that the P2X4 and P2X7 receptors are unlikely to have a significant role in mediating ATP-induced Ca^2+^ responses in Huh-7 cells, thus differing from rat HCC cells [39]. Species difference could be an important factor, and if this is true, cautions need to be exercised in using rodent cells and disease models to elucidate the molecular mechanisms underlying human HCC.

The second interesting finding from the present study is specific expression of the P2Y_11_ receptor in human HCC tissues. A recent study has reported functional expression of the P2Y_2_ receptor in hepatocytes, and its expression was elevated in human primary HCC, HepG2 and BEL-7404 cells [37]. Here, our results indicate the P2Y_11_ receptor is abundantly expressed in human HCC tissues but barely detected in normal liver tissues (Figure 5). This finding brings significant implications. Such HCC-specific P2Y_11_ receptor expression provides a promising disease biomarker, although it is clearly interesting to examine whether there is close association of the P2Y_11_ receptor expression with the severity or grade of human HCC. As discussed below, our study suggests a potential role for the P2Y_11_ receptor in ATP-induced stimulation of cell migration. Further preclinical studies are required to confirm the role of the P2Y_11_ receptor in the regulation of HCC cell migration and metastasis *in vivo*, and if this is true, it is interesting to explore the therapeutic promise of targeting the P2Y_11_ receptor for HCC treatment, as a number of P2Y_11_ receptor selective antagonists have already developed for clinical uses mainly as anti-platelet drugs [14].

Emerging evidence suggests high micromolar concentrations of extracellular ATP at the tumour sites [6, 7]. Such information, when considered together with HCC-specific expression of the P2Y_11_ receptor, raises important questions with regard to the relationship of ATP-induced P2Y_11_-mediated signalling mechanism to the pathogenesis of HCC. In this study, we showed that extracellular ATP up to 100 μM had no effect on Huh-7 cell viability (Supplementary Figure 3) but significantly stimulated cell migration (Figure 6A). As mentioned above, a recent study has reported similar ATP-induced stimulation of cell migration of native human HCC cells and HepG2 and BEL-7404 and attributed the P2Y_2_ receptor to be critical [37]. In contrast, the present study provides evidence to suggest a distinctive or additional mechanism responsible for ATP-induced stimulation of HCC cell migration. The P2Y_11_ receptor agonist NF546 was equally potent and even more potent than ATP in stimulating Huh-7 cell migration (Figure 6). Importantly, treatment with the P2Y_11_ receptor antagonist NF340 completely prevented ATP-induced cell migration (Figure 7A–7B). Therefore, these results support activation of the P2Y_11_ receptor to be the important molecular mechanism for ATP-induced stimulation of Huh-7 cell migration. Intriguingly, there was no effect of ATP or NF546 on cell migration in scratch-induced wound healing assays (data not shown). Such discrepancy in the results may be related to the difference in the mode of cell migration and the way it is assessed. Cancer cell migration requires important regulations of cell cytoskeleton, volume, morphology, cell-to-matrix and cell-to-cell adhesions. Over the recent years, multiple modes of cancer cell migration have been characterized, from single-cell, when cell-to-cell junctions are absent, to collective migration, when cells move as multicellular groups [41]. Two modes on individual cell migration have been described, the “mesenchymal mode”, in which cytoskeletal protrusions and adhesion capabilities are strong, and cells harbour an elongated fibroblast-like morphology, with a rear-to-front lamellopodial cell polarity, focalized cell-matrix adhesions containing integrin clusters and proteolytic activity towards the extracellular matrix (ECM) [42], and the “amoeboid mode”, in which cancer cells show no obvious polarity but a rounded morphology, a weaker adhesion to the substratum, and display high potentials for motility because of their capacity to deform and squeeze inside tissue gaps [43]. Furthermore, as shown in our recent study, these modes of migration can be further unstable and change as the microenvironmental condition changes, resulting in intermediate or mixed phenotypes [44]. Cell migration, assessed in the trans-well assay, strongly depends on the capacity of cells to deform themselves and go through the pores of the inserts and, by contrast, in the wound-healing assay, the ability of cells to form and recycle focal adhesions is critical. Therefore, one could argue that P2Y_11_ activation might induce a type of migration that would be related to the amoeboid mode. In addition, in our experimental conditions of trans-well migration, agonists (ATP or NF546) were added into the lower compartment and might act as chemoattractant, which is not the case in the wound-healing assay. The present study indicates contribution of adenosine receptors in ATP-induced stimulation of cell migration (Figure 7C). Evidently, further studies are required to gain a better understanding of these intriguing observations.

In summary, our study provides strong evidence to show human HCC-specific expression of the P2Y_11_ receptor and its critical role in ATP-induced Ca^2+^ signalling and cell migration. These findings are important for not only understanding of the pathogenesis of HCC but also for identification of disease biomarkers and drug targets in development of new diagnosis and therapeutic approaches to HCC.

## MATERIALS AND METHODS

### Reagents and cell culture

All general chemicals were purchased from Sigma-Aldrich, except those indicated specifically. Phosphate-buffered saline (PBS), Dulbecco's modified Eagle's medium (DMEM), foetal bovine serum (FBS), penicillin-streptomycin, trypsin-EDTA, pluronic acid F-127 were from Life Technology, and MRS2768, 5-BDBD, NF546 and NF340 from Tocris Bioscience. MRS2768 was also obtained from Santa Cruz. Huh-7 cells were kindly provided by Prof M Harris (University of Leeds, UK).

Huh-7 and HepG2 cells were maintained in DMEM supplemented with 10% heat inactivated FBS, 100 U/ml penicillin and 100 μg/ml streptomycin at 37°C and 5% CO_2_, and passaged when cells reached 70% confluency. Huh-7 and HepG2 cells were transfected with 15 nM small interfering RNA (siRNA) targeting the *P2YR11* gene with the following sequences (siP2Y11): forward 5’-CCUGCUGGGCAGCGUCAUC(TT)-3’ and reverse 5’-GAUGACGCUGCCCAGCAGG(TT)-3’. Irrelevant sequences, not targeting any known gene were used as control sequences (siCTL): forward, 5’-GCCGACCAAUUCACGGCCG(TT)-3’ and reverse, 5’-CGGCCGUGAAUUGGUCGGC(TT)-3’. These sequences were produced by Sigma-Aldrich. Transfection was performed with Lipofectamine RNAi max (Invitrogen) according to the manufacturer's instructions, and cells were used 72 hr after transfection.

### [Ca^2+^]_i_ measurement

Agonist-induced changes in the [Ca^2+^]_i_ was monitored using Fura-2/AM dye and FLEXstation III (Molecular Device). Cells were seeded onto a 96-well assay plates (Greiner Bio-one) at a density of 2-5x10^4^ cells per well and incubated in culture medium overnight. Cells were loaded with 2 μM Fura-2/AM and 0.4% pluronic acid F-127 (Molecular Probes) in standard buffer solution (SBS: 147 mM NaCl, 2 mM KCl, 1.5 mM CaCl_2_, 1 mM MgCl_2_, 10 mM HEPES and 13 mM glucose 13, pH 7.3) at 37°C for 1 hr, and after washing, maintained in SBS at 37°C for 30 min. F340/F380, the ratio of the fluorescence intensity excited alternatively by 340 nm and 380 nm and emitted at 510 nm, was used to indicate the [Ca^2+^]_i_. ΔF340/F380 is agonist-induced change in F340/F380. Agonist was added after the baseline was established. Antagonists were added 5 min before addition of agonist. ATP concentration-response curve was constructed by expressing ATP-induced ΔF340/F380 as percentage of ΔF340/F380 induced by 100 μM ATP (Figure 1B) and least squared fit to Hill equation: ΔF340/F380=100/(1+(EC_50_/[agonist])^n^), where EC_50_ is the agonist concentration evoking half of the maximal Ca^2+^ response, and n is Hill coefficient. Data fit was carried out using Origin software.

### Immunocytochemistry

Huh7 cells were seeded on coverslips and incubated in culture medium overnight. Cells were fixed with 4% paraformaldehyde for 10 min and permeabilized with 0.2% Triton X-100 in phosphate buffer saline (PBS-T) for 10 min, and blocked with 5% goat serum or bovine serum albumin in PBS for 1 hr. Cells were then incubated with primary rabbit anti-P2X7 or anti-P2Y_11_ antibody (Almone) at a dilution of 1: 50 overnight at 4°C and, after extensive washing in PBS-T, were incubated with FITC-conjugated anti-rabbit IgG secondary antibody (Sigma) at a dilution of 1:1000 for 1 hr at room temperature. After washing in PBS-T, coverslips were rinsed in water, dried on tissue papers, and mounted inversely on a glass slide with a small drop of anti-fade mounting medium containing 4’,6-diamidino-2-phenylindole (DAPI). Fluorescent images were captured using an EVOS Cell Imaging System (Thermo Fisher Scientific) and images were merged by Image J software.

Human samples of normal liver and hepatocellular carcinoma were obtained from the tumour biobank of the University-Hospital of Tours, declared to the French Ministry of Research (N°DC2008-308). Briefly, tissues were fixed in formalin, paraffin included, and cut in 5 μm sections. Slides were deparaffinized, rehydrated, and heated in citrate buffer pH 6.0 for antigenic retrieval. Slides were incubated with rabbit anti-P2Y_11_ antibody at 1: 200 for 1 hr. Immunohistochemistry was performed using the streptavidin-biotin-peroxidase method with diaminobenzidine as the chromogen (Kit LSAB, Dakocytomation). Slides were finally counterstained with haematoxylin. Negative control was obtained after omission of the primary antibody.

### Western blotting

Cells were washed with PBS and lysed in presence of a lysis buffer (20 mM Tris, pH 7, 150 mM NaCl, 1 mM MgCl_2_, 1 mM CaCl_2_), containing 1% Triton X-100 and a cocktail of protease inhibitor (Sigma-Aldrich). Cell lysates were cleared by centrifugation at 10,000 x g for 10 min. Western blotting experiments were performed according to standard protocols. Total protein concentrations were determined using a Pierce® BCA Protein Assay Kit (Fisher Scientific). Protein sample buffer was added and the samples were boiled at 100°C for 3 min. Total protein samples were electrophoretically separated by sodium dodecyl sulphate-polyacrylamide gel electrophoresis in 10% gels, and transferred to polyvinylidene fluoride membranes (Millipore). P2Y_11_ proteins were detected using anti-P2Y_11_ antibody at a dilution of 1:1,000 and horseradish peroxidase-conjugated goat anti-rabbit IgG secondary antibody at 1:5,000 (TebuBio). HSC70 protein was detected as a sample loading control using anti-HSC70 mouse antibody at 1:30,000 (TebuBio) and HRP-conjugated anti-mouse-IgG secondary antibodies at 1:2,000 (TebuBio).

### Cell migration assay

Trans-well cell migration assays were carried out in 24-well plates with polyethylene terephthalate membrane cell culture inserts containing 8-μm trans-well pores (BD Biosciences) as described in our recent study [45]. The upper compartment was seeded with 1 x 10^5^ cells, and both the upper and lower compartments were filled with DMEM supplemented with 10% FBS, which represent the control conditions. Agonist was added to the medium in the lower chamber, and antagonist was added into the upper chamber at the same time as or 30 min before addition of agonist. After incubation at 37°C and 5% CO_2_ for 24 hr, cells were fixed with 4% paraformaldehyde and stained with 0.05% crystal violet for 30 min at room temperature. Cells in 9 different areas of each insert were imaged using an ECLIPSE TE2000-U microscope (Nikon) and counted using Image J software. For meaningful comparisons between different experiments, cell migration was presented by expressing the migrated cell number as % of that under control conditions.

### Cell death assay

Propidium iodide (PI) staining cell death assays were carried out to examine the effects of ATP on cell viability, cells were seeded on 24-well plated at 1x 10^4^ cells per well and incubated in culture medium without or with supplementation of ATP for 24 hr. Cells were stained with 5 μg/ml PI and 1 μg/ml Hoechst for 30 min. Images were captured using an EVOS Cell Imaging System (Thermo Fisher Scientific).

### Data presentation and statistical analysis

All data are presented as mean ± standard error of mean (S.E.M.), where appropriately. Statistical analysis was carried out using Student's *t*-test to compare two groups one-way ANOVA and Tukey post hoc tests to compare more than two groups using Origin software, with *p* < 0.05 being indicative of significance.

## SUPPLEMENTARY MATERIALS FIGURES


