# Supplementary material for: Carcinoma-specific expression of P2Y11 receptor and its contribution in ATP-induced purinergic signalling and cell migration in human hepatocellular carcinoma cells
Source: Oncotarget. 2017 Mar 14;8(23):37278–90. doi: 10.18632/oncotarget.16191 (PMC5514908; doi:10.18632/oncotarget.16191)
Supplement: Supplementary file 1 [file oncotarget-08-37278-s001.pdf]

Carcinoma-specific expression of $P2Y_{11}$ receptor and its contribution in ATP-induced purinergic signalling and cell migration in human hepatocellular carcinoma cells

SUPPLEMENTARY MATERIALS

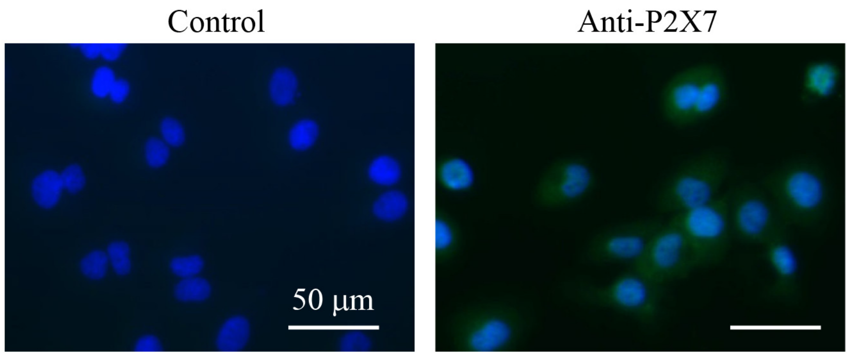

Supplementary Figure 1: Immunostaining of the P2X7 receptor in Huh-7 cells. Representative confocal images showing control cells stained only with the secondary antibody (left panel) and cells stained with primary anti-P2X7 antibody and the secondary antibody (right panel). Cells were countstained with DAPI. Similar results were observed in two independent experiments.

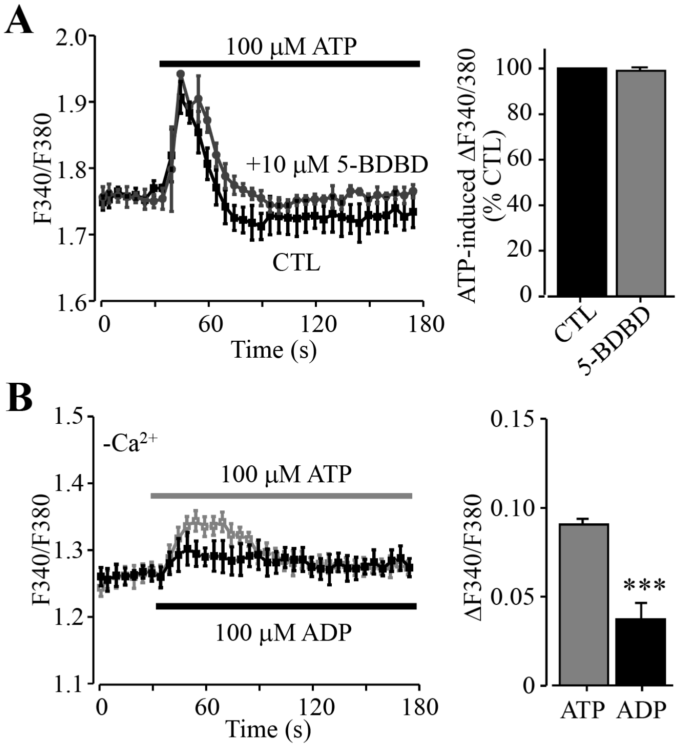

Supplementary Figure 2: Ca^{2+} responses to P2X or P2Y agonists in Huh-7 cell. **(A)** *Left*, representative recordings of 100 μM ATP-induced Ca^{2+} responses in control cells or cells pre-treated with 10 μM 5-BDBD, with six wells of cells for each case. *Right*, summary of ATP-induced peak increase in the $[Ca^{2+}]_i$ in control and 5-BDBD-treated cells, expressed as % of that in control cells, from twelve wells from two independent experiments. **(B)** *Left*, representative recordings of the Ca^{2+} responses induced by 100 μM ADP (black) and ATP (grey) in extracellular Ca^{2+}-free solutions with six wells of cells. *Right*, summary of the peak increase in the $[Ca^{2+}]_i$.***, $p < 0.001$.

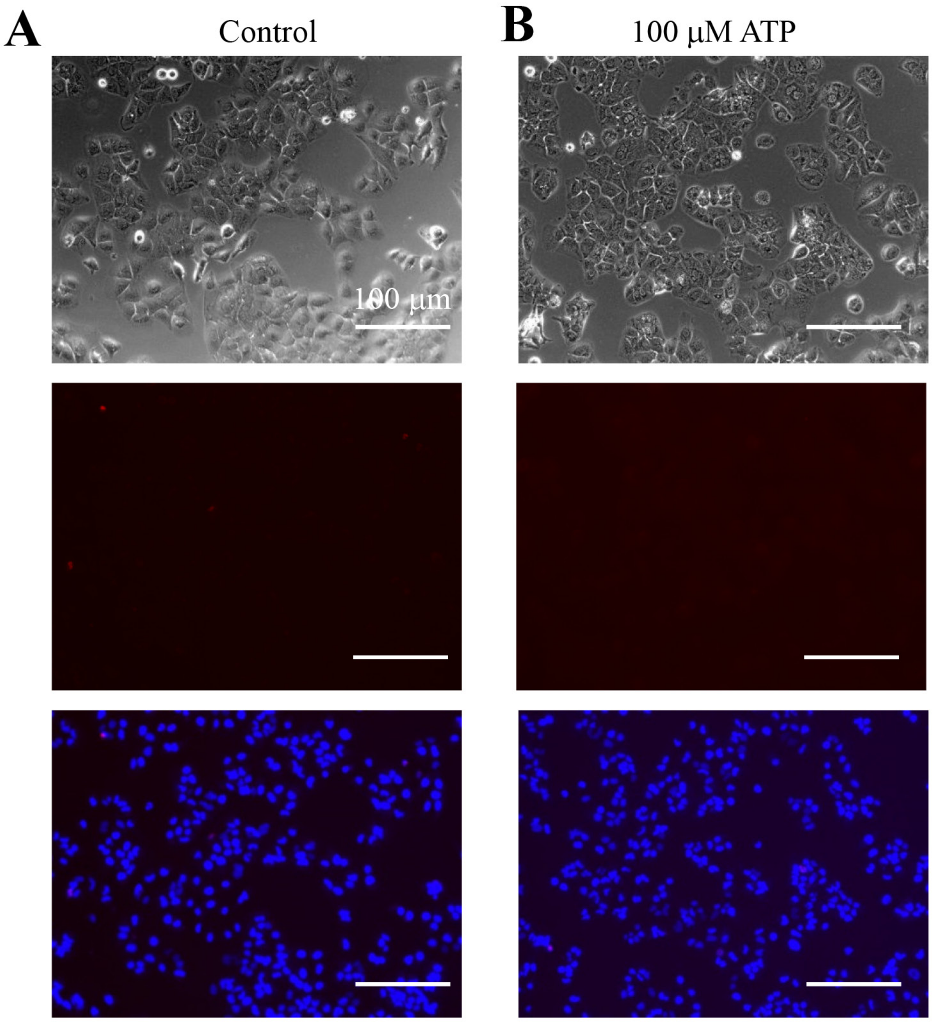

Supplementary Figure 3: No effect of ATP on Huh-7 cell survival. Representative bright field (top) and fluorescent images showing PI staining (middle) and merged images (bottom) showing PI and Hoechst staining under control conditions **(A)** and in the presence of 100 µM ATP for 24 hr **(B)** from four wells of cells for each condition.
